# Supplementary material for: RNA-mediated ribonucleoprotein assembly controls TDP-43 nuclear retention
Source: PLoS Biol. 2024 Feb 29;22(2):e3002527. doi: 10.1371/journal.pbio.3002527 (PMC10931518; doi:10.1371/journal.pbio.3002527)
Supplement: S1 Fig — (PDF) [file pbio.3002527.s001.pdf]

Supporting Figure 1

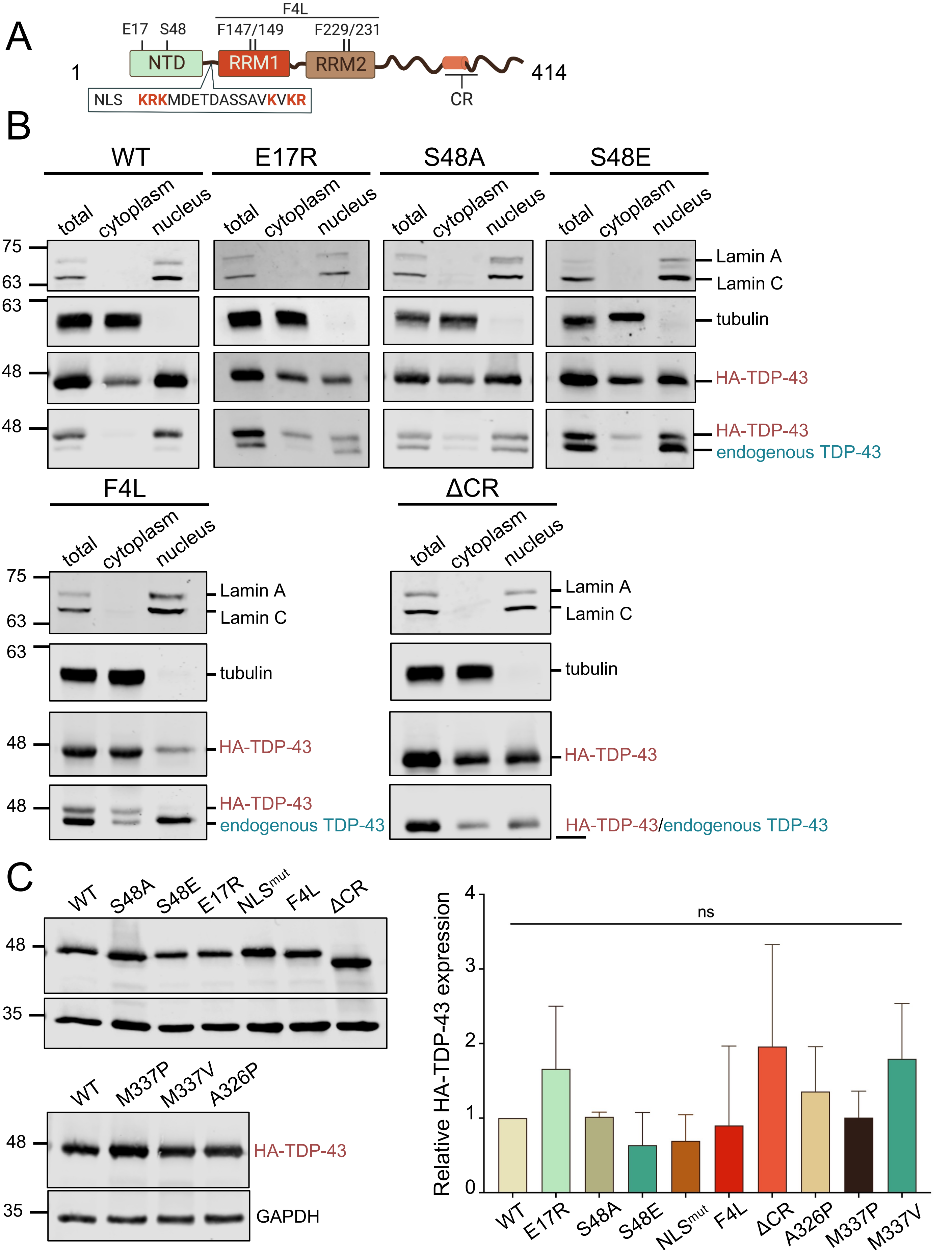

Supporting Figure 1. Defects in TDP-43 self-assembly and RNA binding decrease nuclear localization. A) TDP-43 domain organization highlighting targeted mutation sites to inhibit N-terminal domain (NTD) oligomerization, RNA binding and C-terminal assembly through deletion of the conserved α-helical region (CR) in the C-tail (a.a. 316-343). The basic residues (red) in the nuclear localization signal (NLS) were substituted to generate NLS^{mut}, used as control. B) TDP-43 levels in nuclear and cytoplasmic compartments were compared after biochemical fractionation. The immunoblots detect HA-TDP-43 and endogenous TDP-43 in total, cytoplasmic and nuclear fractions of the stable $HEK293^{HA\text{-}TDP\text{-}43}$ cells. Lamin A/C and tubulin were used as nuclear and cytoplasmic controls, respectively. Source images can be found in S1 Raw Images. C) Relative HA-TDP-43 expression in each cell line calculated from total cell lysate, compared to WT. GAPDH was used as loading control. The plot shows Mean $\pm$ SD is for 4 independent replicates. ns, non-significant differences according to one-way ANOVA with Tukey's test. Source data can be found S1 Data.
