# Supplementary material for: RNA-mediated ribonucleoprotein assembly controls TDP-43 nuclear retention
Source: PLoS Biol. 2024 Feb 29;22(2):e3002527. doi: 10.1371/journal.pbio.3002527 (PMC10931518; doi:10.1371/journal.pbio.3002527)
Supplement: S2 Fig — (PDF) [file pbio.3002527.s002.pdf]

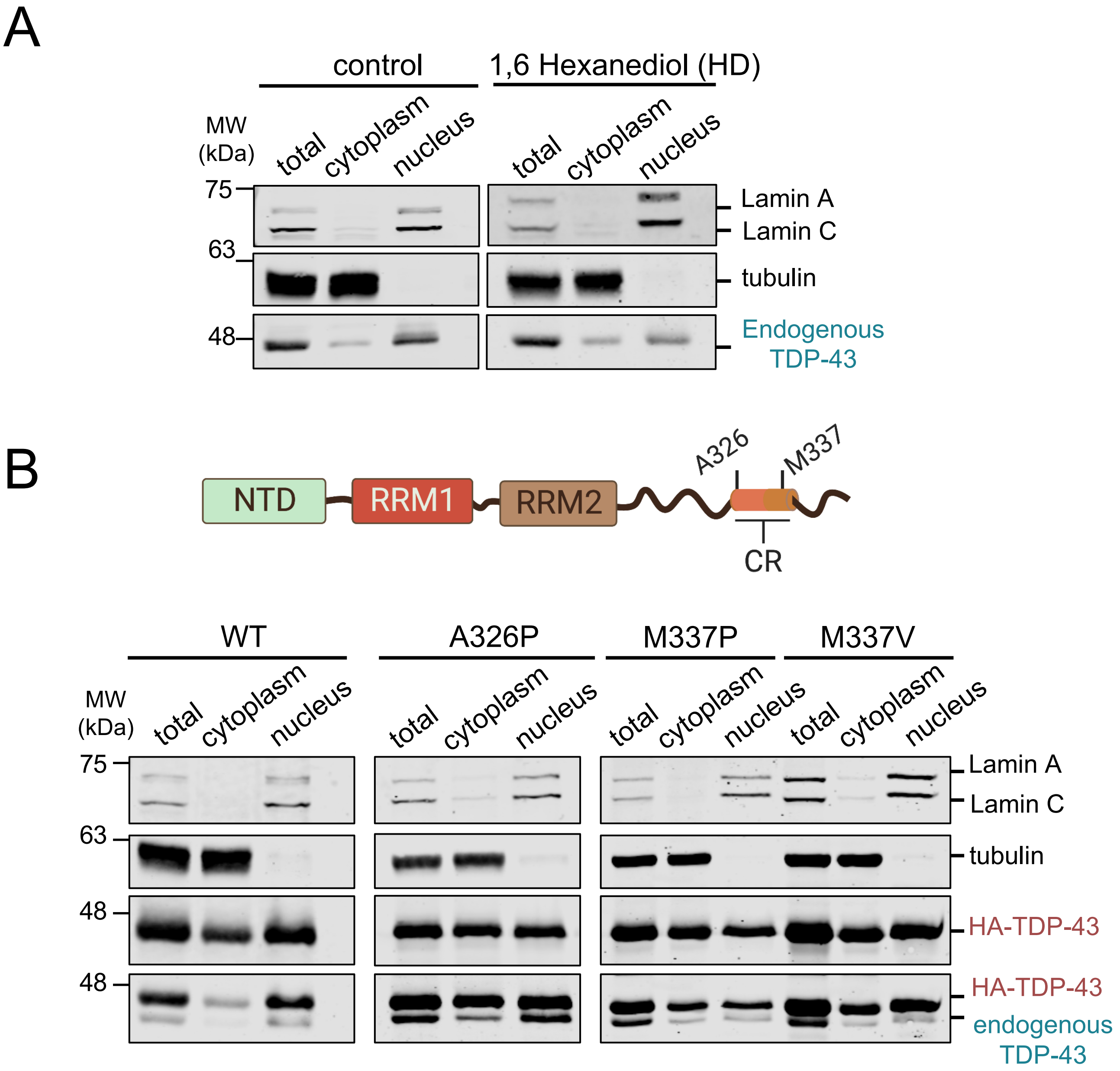

Supporting Figure 2. Inhibition of phase separation disrupts TDP-43 nuclear retention. A) Biochemical fractionation of HEK293 cells treated with 1,6 hexanediol (HD, 5% for 5 min), or non-treated cells into nuclear and cytoplasmic compartments. Immunoblots probed for endogenous TDP-43 in total, nuclear and cytoplasmic fractions. Lamin and tubulin are nuclear and cytoplasm controls, respectively. B) Immunoblots of total, nuclear and cytoplasmic fractions from $HEK293^{HA\text{-}TDP\text{-}43}$ cells expressing phase separation-defective mutants, including ALS-linked M337V. Source images can be found in S1 Raw Images.
