# Supplementary material for: RNA-mediated ribonucleoprotein assembly controls TDP-43 nuclear retention
Source: PLoS Biol. 2024 Feb 29;22(2):e3002527. doi: 10.1371/journal.pbio.3002527 (PMC10931518; doi:10.1371/journal.pbio.3002527)
Supplement: S3 Fig — (PDF) [file pbio.3002527.s003.pdf]

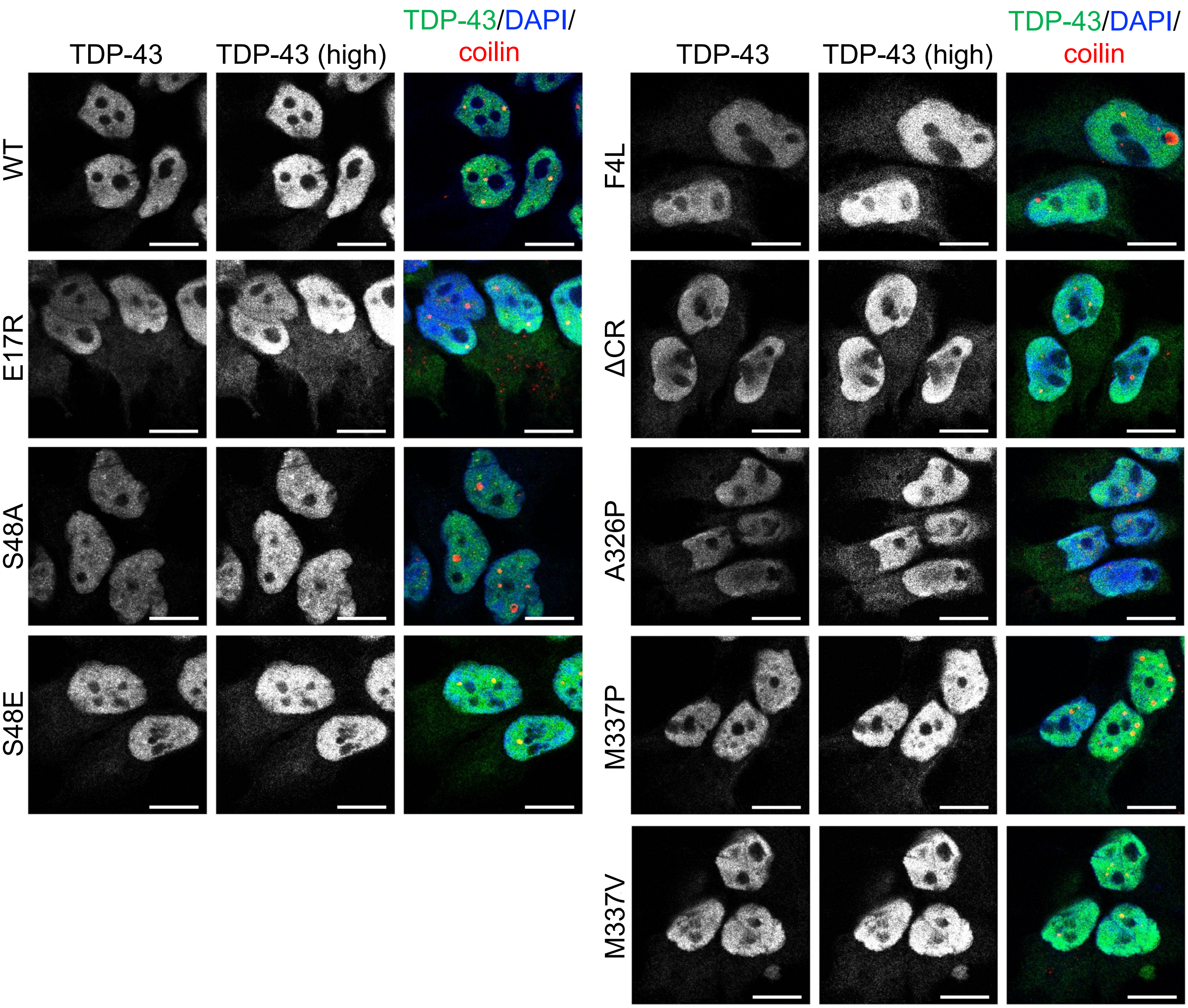

Supporting Figure 3. Cellular distribution of WT and mutant TDP-43 as seen by microscopy. Immunofluorescence of HEK293$^{HA\text{-}TDP\text{-}43}$ cells expressing HA-tagged WT and indicated mutant TDP-43 after 48 h of Tet induction, as seen by confocal microscopy. The Cajal body marker coilin and HA antibodies were used for detection. Scale, 10 µm.
