# Supplementary material for: RNA-mediated ribonucleoprotein assembly controls TDP-43 nuclear retention
Source: PLoS Biol. 2024 Feb 29;22(2):e3002527. doi: 10.1371/journal.pbio.3002527 (PMC10931518; doi:10.1371/journal.pbio.3002527)
Supplement: S4 Fig — (PDF) [file pbio.3002527.s004.pdf]

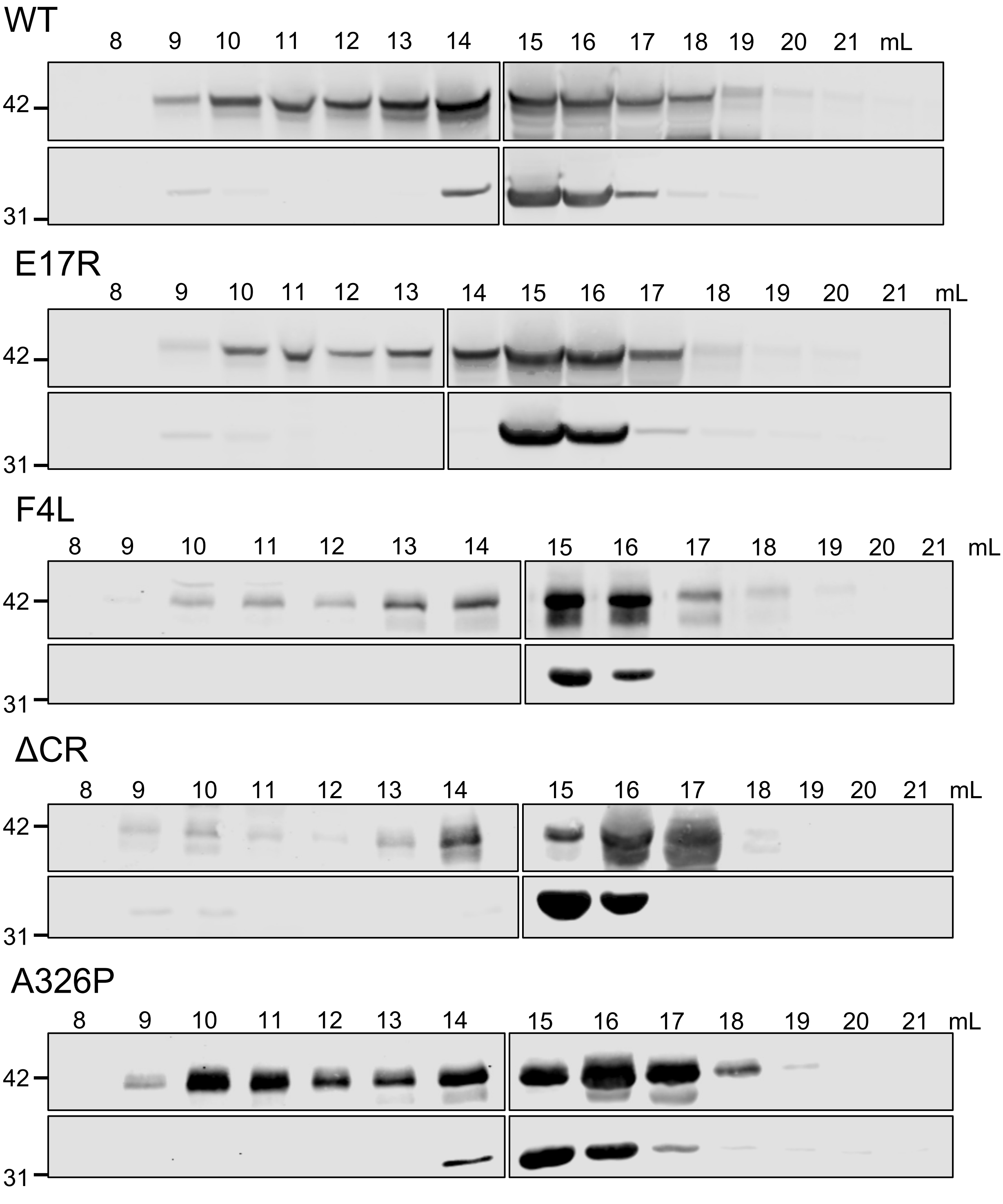

Supporting Figure 4. TDP-43 macromolecular complex distribution in total cell lysate. Immunoblot detecting HA-TDP-43 levels in fractions eluted from gel filtration size exclusion chromatography of $HEK293^{HA\text{-}TDP\text{-}43}$ cell lysate. Membranes were probed with HA antibody and GAPDH as control. Source images can be found in S1 Raw Images.
