# Supplementary material for: RNA-mediated ribonucleoprotein assembly controls TDP-43 nuclear retention
Source: PLoS Biol. 2024 Feb 29;22(2):e3002527. doi: 10.1371/journal.pbio.3002527 (PMC10931518; doi:10.1371/journal.pbio.3002527)
Supplement: S1 Table — (PDF) [file pbio.3002527.s005.pdf]

Supporting Table 1. Oligonucleotides used in these studies

DNA	Sequence
TDP43_A326P Fw	5'-CCAGCCATGATGGCTGCCCCACAGGCAGCACTACAGAGCAGTTGG-3'
TDP43_A326P Rv	5'-CCAACTGCTCTGTAGTGCTGCCTGTGGGGCAGCCATCATGGCTGG-3'
TDP43_M337P Fw	5'-CAGAGCAGTTGGGGTATGCCAGGCATGTTAGCCAGCCAG -3'
TDP43_M337P Rv	5'-CTGGCTGGCTAACATGCCTGGCATACCCCAACTGCTCTG-3'
RNA	RNA sequence (r) with modifications:<br>m: 2' O-methyl; *: phosphorothioate; /3Bio/: 3'-biotin
(CA)6-biotin	mC*mA*mC*mA*mC*mA*mC*mA*mC*mA*mC*rA /3Bio/
(GU)6-biotin	mG*mU*mG*mU*mG*mU*mG*mU*mG*mU*mG*rU /3Bio/
