# Supplementary material for: RNA-mediated ribonucleoprotein assembly controls TDP-43 nuclear retention
Source: PLoS Biol. 2024 Feb 29;22(2):e3002527. doi: 10.1371/journal.pbio.3002527 (PMC10931518; doi:10.1371/journal.pbio.3002527)
Supplement: S1 Raw Images — (PDF) [file pbio.3002527.s007.pdf]

PLOS Biology Submission: PBIOLOGY-D-23-02154R1

## 1B_raw_images

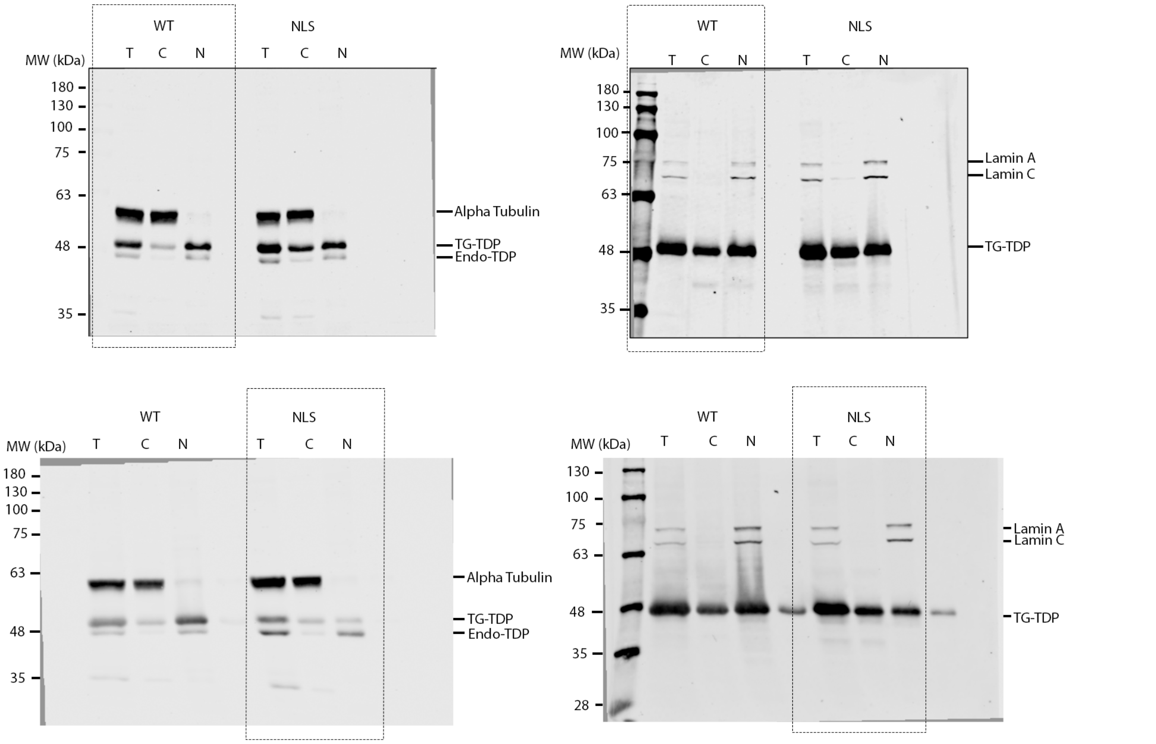


T: Total Fraction; C: Cytoplasmic Fraction; N; Nuclear Fraction

## 5A_raw_images

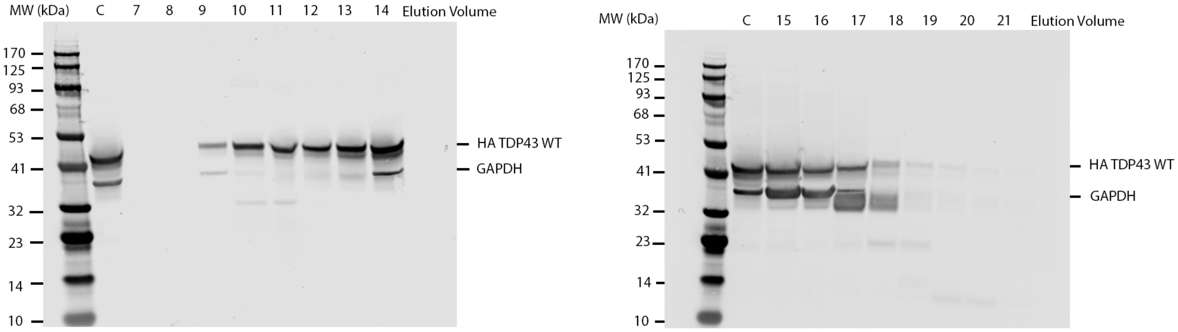


MW: Molecular weight marker; C: Control Sample used to normalize the band intensities of two different membranes

S1B_raw_images

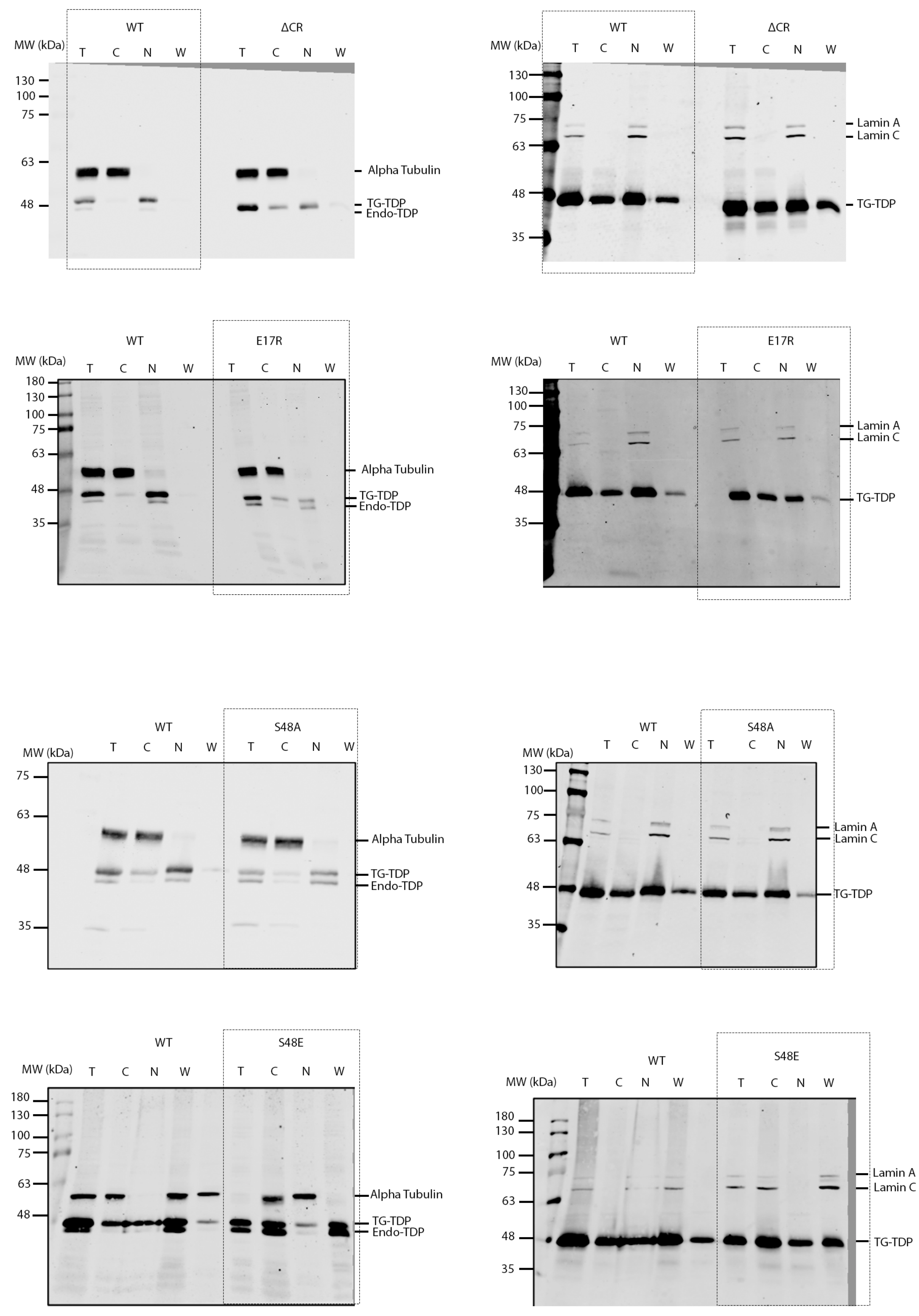

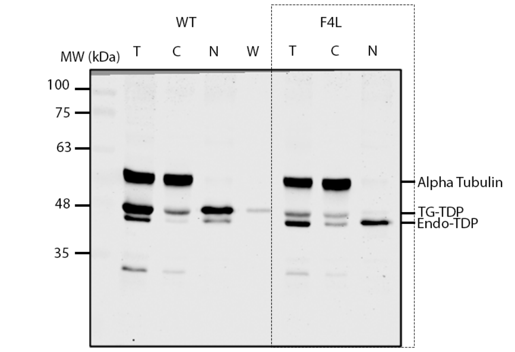


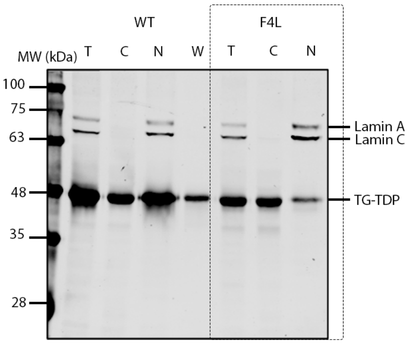


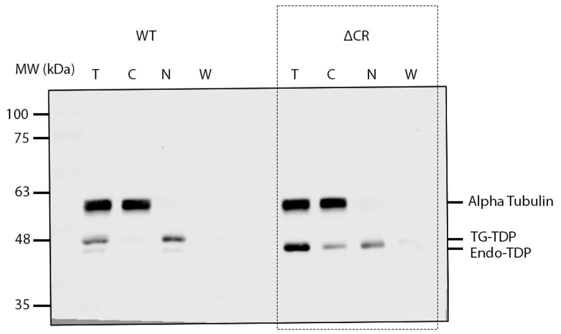


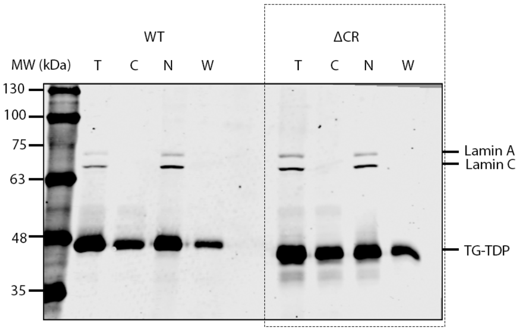


T: Total Fraction; C: Cytoplasmic Fraction; N; Nuclear Fraction; W: Washed Fraction

# S2A_raw_images

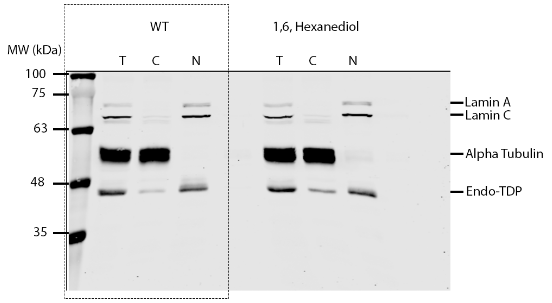


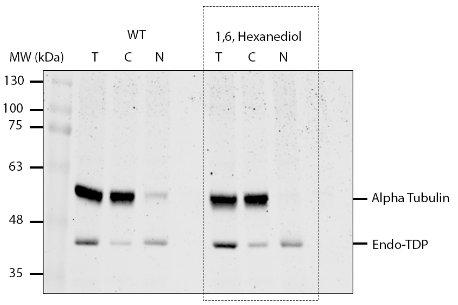


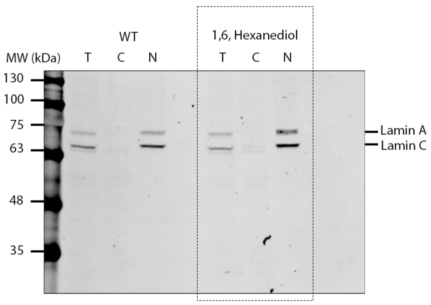


T: Total Fraction; C: Cytoplasmic Fraction; N; Nuclear Fraction

# S2B_raw_images

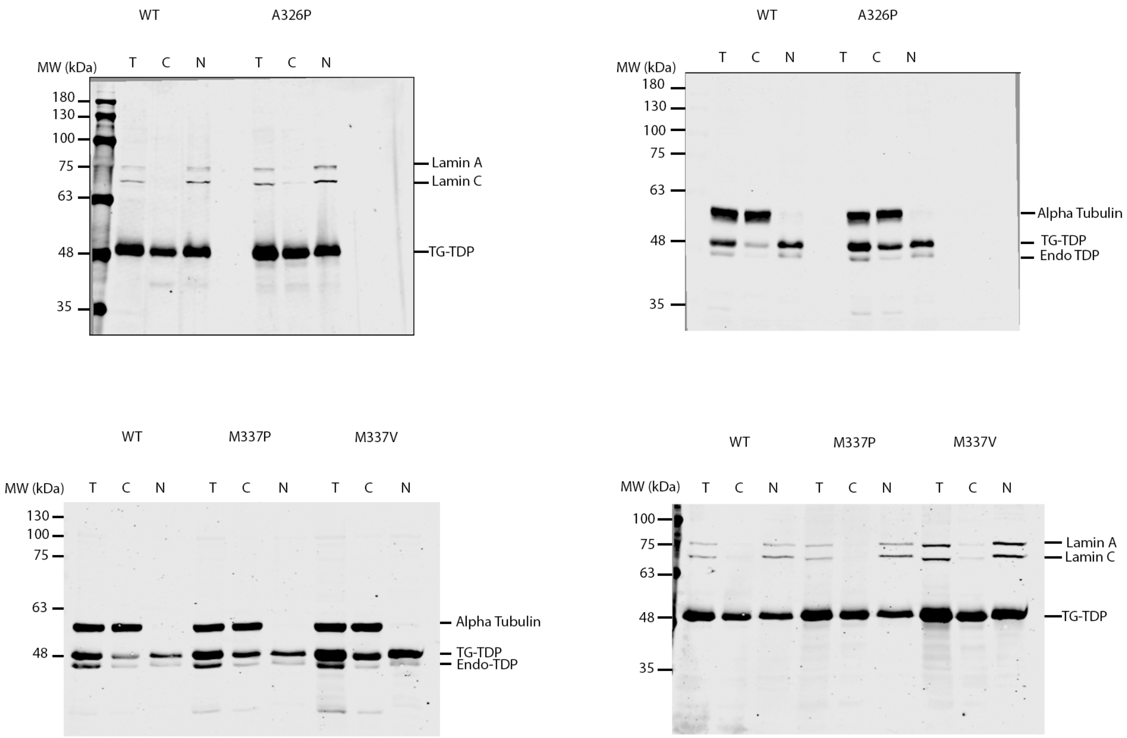


T: Total Fraction; C: Cytoplasmic Fraction; N; Nuclear Fraction

# S3_raw_images

# WT

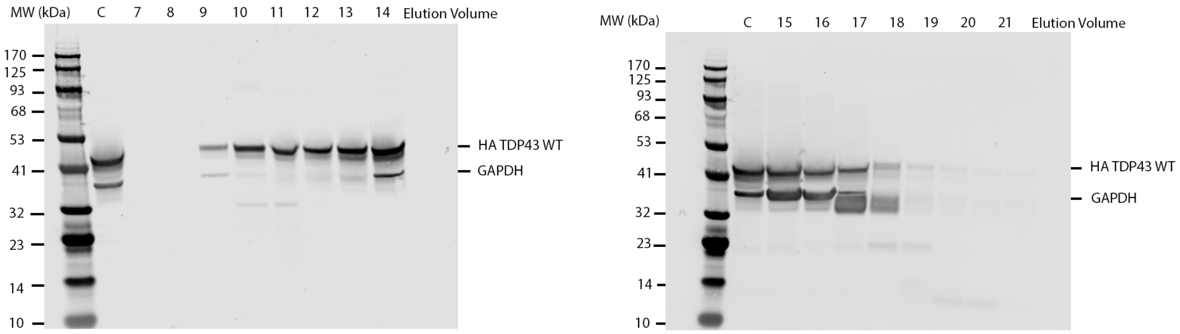


MW: Molecular weight marker; C: Control Sample used to normalize the band intensities of two different membranes

# E17R

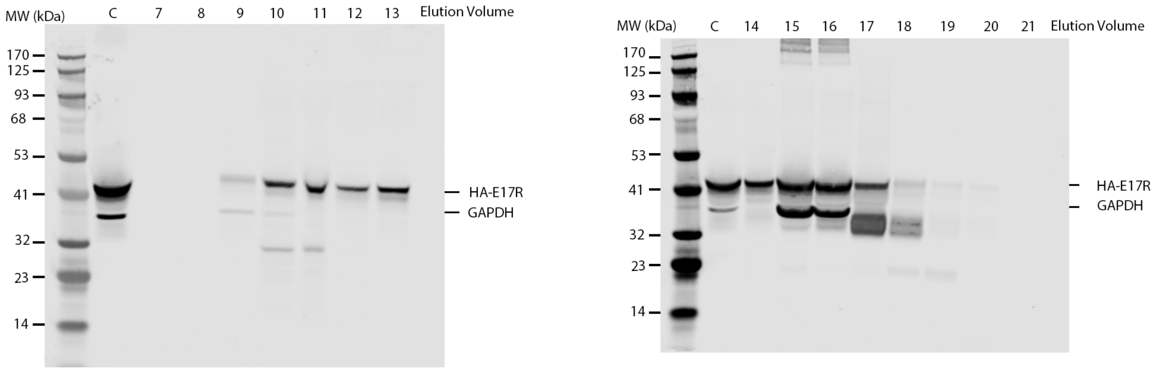
MW (kDa)
C 7 8 9 10 11 12 13 Elution Volume
170
125
93
68
53
41
32
23
14
HA-E17R
GAPDH
MW (kDa)
C 14 15 16 17 18 19 20 21 Elution Volume
170
125
93
68
53
41
32
23
14
HA-E17R
GAPDH

# F4L

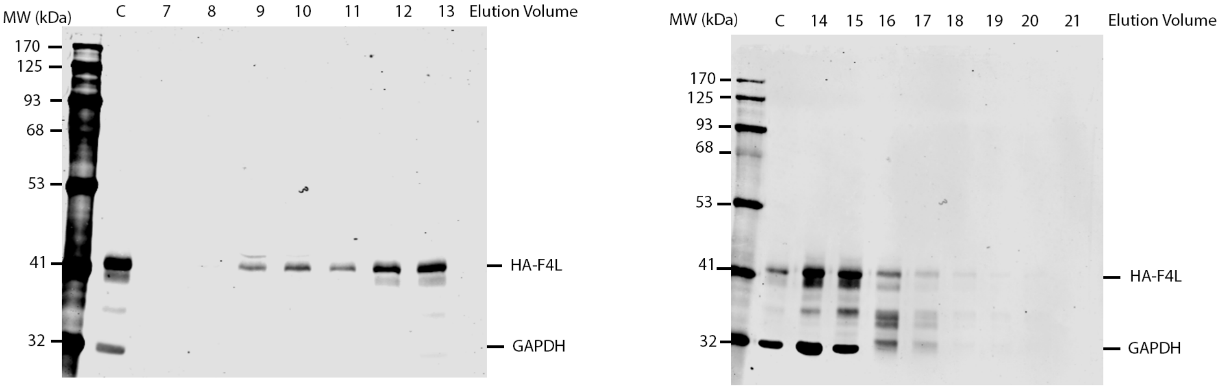
MW (kDa)
C 7 8 9 10 11 12 13 Elution Volume
170
125
93
68
53
41
32
HA-F4L
GAPDH
MW (kDa)
C 14 15 16 17 18 19 20 21 Elution Volume
170
125
93
68
53
41
32
HA-F4L
GAPDH

# ΔCR

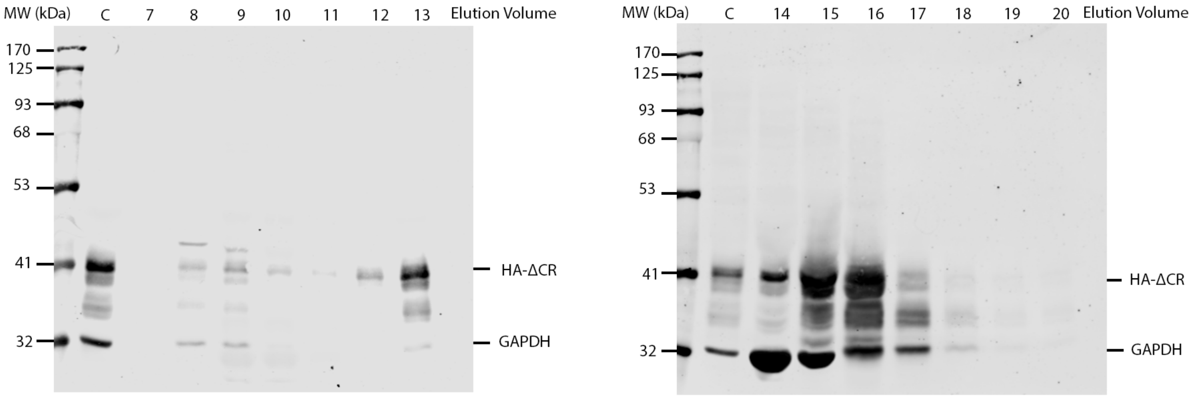
MW (kDa)
C 7 8 9 10 11 12 13 Elution Volume
170
125
93
68
53
41
32
HA-ΔCR
GAPDH
MW (kDa)
C 14 15 16 17 18 19 20 Elution Volume
170
125
93
68
53
41
32
HA-ΔCR
GAPDH

# A326P

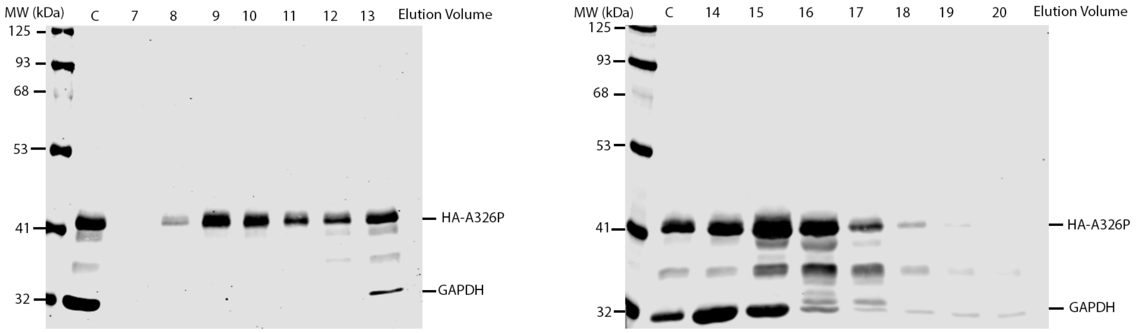
MW (kDa)
C
7
8
9
10
11
12
13
Elution Volume
125
93
68
53
41
32
HA-A326P
GAPDH
MW (kDa)
C
14
15
16
17
18
19
20
Elution Volume
125
93
68
53
41
32
HA-A326P
GAPDH
